# Numerical Simulation of Particle Distribution in Capillary Membrane during Backwash

**DOI:** 10.3390/membranes3040249

**Published:** 2013-09-27

**Authors:** Hussam Mansour, Anik Keller, Rolf Gimbel, Wojciech Kowalczyk

**Affiliations:** 1 Department of Mechanics and Robotics, University of Duisburg-Essen, Lotharstr. 1, 47057 Duisburg, Germany; E-Mail: wojciech.kowalczyk@uni-due.de; 2 Department of Process Engineering and Water Technology, University of Duisburg-Essen, Bismarckstr. 90, 47057 Duisburg, Germany; E-Mails: anik.keller@uni-due.de (A.K.); rolf.gimbel@uni-due.de (R.G.); 3 IWW Water Research Institute, Moritzstr. 26, 45476 Mülheim an der Ruhr, Germany

**Keywords:** capillary membranes, backwash, numerical simulation, multiphase flow, ultrafiltration, particle distribution

## Abstract

The membrane filtration with inside-out dead-end driven UF-/MF- capillary membranes is an effective process for particle removal in water treatment. Its industrial application increased in the last decade exponentially. To date, the research activities in this field were aimed first of all at the analysis of filtration phenomena disregarding the influence of backwash on the operation parameters of filtration plants. However, following the main hypothesis of this paper, backwash has great potential to increase the efficiency of filtration. In this paper, a numerical approach for a detailed study of fluid dynamic processes in capillary membranes during backwash is presented. The effect of particle size and inlet flux on the backwash process are investigated. The evaluation of these data concentrates on the analysis of particle behavior in the cross sectional plane and the appearance of eventually formed particle plugs inside the membrane capillary. Simulations are conducted in dead-end filtration mode and with two configurations. The first configuration includes a particle concentration of 10% homogeneously distributed within the capillary and the second configuration demonstrates a cake layer on the membrane surface with a packing density of 0.6. Analyzing the hydrodynamic forces acting on the particles shows that the lift force plays the main role in defining the particle enrichment areas. The operation parameters contribute in enhancing the lift force and the heterogeneity to anticipate the clogging of the membrane.

## 1. Introduction

The application of ultrafiltration (UF) capillary membranes in water treatment increases exponentially and has become more and more important in industrial fluid separations. It is a promising technology widely employed to enhance the purity and safety of drinking water [1] since it offers the advantages of high efficiency with the use of mainly physical processes, *i.e.*, in many cases without additives of chemicals and relatively low operational costs and energy consumption [2]. However, the process is limited by membrane fouling caused by physical and/or chemical interactions between the membrane and particulate matter existing in the feed water [3]. The accumulation of these particles during filtration forms a cake layer, which creates additional resistance and contributes to the reduction of permeability of the membrane. The formation of this layer was studied in the presence of adhesive forces on the membrane surface and on different distributed membrane pore sizes [4]. A new model was developed for crossflow filtration of polydisperse particles [5] to investigate the factors influencing the cake formation during the filtration process and the cake growth on non-uniform permeable membranes [6,7]. CFD simulations allow better insight into transport mechanisms of particles and consequently offer numerous improvement possibilities on the membrane process including new membrane materials and configurations, determining operation conditions for optimum selectivity and defining the permeate flux to minimize fouling [8]. The total permeate flux of crossflow microfiltration membranes is predicted by 3-dimensional CFD simulations to obtain the pressure distribution upon the membrane surface [9]. The accumulation of particles on porous surfaces during crossflow filtration is described by modeling the colloidal phase transition into condensed phase [10,11]. Coupling Navier-Stokes and Darcy equations in crossflow tubular membrane filtration based on finite difference model provides characterization of the flow behavior and contributes to a better understanding of the growth rate of the polarization boundary layer along the tubular membrane [12–14] and the strength and weakness of this coupling is discussed as well [15]. For 3-dimensional crossflow finite element models for different suspended particles, CFD simulation is developed in order to predict the velocity field, pressure drop and particle concentration along a hollow fiber for an ultrafiltration system and to provide information about the overall resistance of the membrane and shear forces [16]. Many techniques have been extensively investigated and developed to increase the filtration process accuracy and performance. The most common technique is backwash. It is an effective method for removing particle cakes and recovering membrane properties [17]. A periodic backwash lifts off the deposited cake and flushes it out of the capillary membrane for the backwash mode outside-in. Many experimental investigations have been performed to improve the membrane cleaning efficiency and to evaluate the potential of air bubbling injection [18–21]. A new controller system was introduced to achieve an optimal backwash duration [22]. Moreover, the adhesive forces and operating conditions were studied on capillary membranes driven in dead-end mode during backwash [4,23].

Numerical simulation of membrane backwash is a powerful tool to increase the understanding of this process but until now some aspects are still lacking for elucidation and optimization. From the numerical point of view minor effects have been spent in investigating backwash process and the affecting parameters on its performance. The objective of this study is on the one hand to highlight the function of the exerted forces on the particles inside capillary membranes operated with dead-end outside-in mode during backwash, and on the other hand to analyze the velocity pattern followed by the demonstration of particle enrichment areas according to different initial particle distributions, particle sizes and water flow rates.

The concept of the whole project contains both numerical and experimental aspects. In the current paper we focused on the theoretical approach and first of all on the development and proof of the numerical multiphase model. Since the numerical investigations have a principal character aiming at the analysis of general fluid mechanical phenomena in a capillary, a direct comparison with experiments is not given. Nevertheless, the first observations and evaluations show some qualitative similarities according to the fluid flow and the behavior of particles. Additionally, some further information from the experiment was applied as initial condition in the numerical model, *i.e.*, thickness of the particle deposited layer and packing density as well as the flux at inlet. Moreover, the numerically calculated pressure drop in the capillary membrane was validated by the experiment. Further experimental validation of a numerical model will concentrate on an analysis of a particle distribution that is measured at the outlet of the capillary membrane at different phases of the process. The results of the significant stronger linking of simulations and experiments will be addressed in subsequent studies.

## 2. Methods

### 2.1. Experimental Section

**Figure 1:**
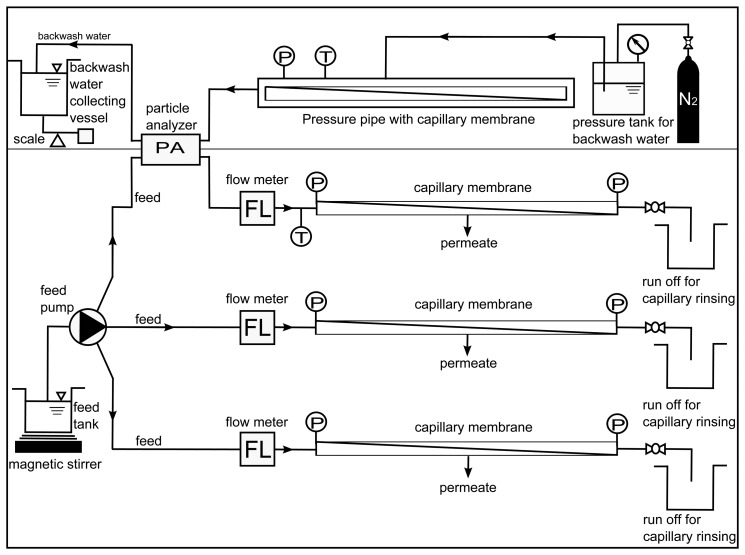
Schematic representation of the filtration and backwash device.

For the filtration experiments both hard (silica powder) (400 mg L^−1^) and soft (baker's yeast cells) particles (50 mg L^−1^) are chosen as additives to the feed in order to cause inorganic and organic particulate membrane fouling. Furthermore, spherical polystyrene particles (100 mg L^−1^) with well-defined size distribution are applied. The filtration device illustrated in Figure 1 (lower part) can generate simultaneously fouling layers in up to three single commercial UF capillary membranes under the same dead-end conditions. The device is operated for 1 liter of the particle suspension at constant flux of 180 L m^−2^ h^−1^ recording temperature (T) and pressure (P). The flow rate and additionally the density of the suspensions are recorded by high sensitive coriolis flowmeters (FL).

A particle analyzer (PA) is implemented in one feed line to monitor the particle shape and size distribution. With this setup the velocity, size and morphology of single particle (bigger than 1 μm) and eventually formed aggregates can be recorded and characterized. The backwash section (upper part of Figure 1) is designed to investigate the removal of particles and fouling layers out of the capillary membranes. During backwash the water is forced through the capillary wall in outside-in direction by applying either constant pressure profiles (1, 2, 3 or 4 bar) or linear pressure function (from 1 to 4 bar). Pressure (P) and backwash flux are monitored continuously. To observe particles and agglomerates flushed out of the capillary the particle analyzer (PA) is implemented. Additionally, the capillary membrane was divided into many segments in which the structure of the deposited layer was analyzed by microscope.

### 2.2. Numerical Simulation

#### 2.2.1. Model Description

Numerical simulation was performed to describe the flow phenomena and motion of particles in the capillary. This study is based on two phase Euler-Euler model for solving the multiphase flow problem. The fluid is simulated as a continuous phase whereas the particles are simulated as a dispersed phase. The continuity (1) and momentum (2) equations are solved with the twoPhaseEulerFoam solver which is based on a finite volume method implemented in free open source CFD framework OpenFOAM-2.0.0 (OpenCFD Ltd.). The continuous phase has the physical properties of pure water, density of 1000 kg m^−3^ and a kinematic viscosity of 1×10^−6^ m^2^ s^−1^. The particles are considered as non-deformable spheres with a constant density of 1050 kg m^−3^. Reynolds number *Re* of 200–700 related to the capillary diameter, maximum velocity and kinematic viscosity of the fluid indicates a laminar flow inside the capillary.

#### 2.2.2. Governing Equations

The basic equations of the multiphase modeling assume an incompressible fluid with the corresponding conservation of mass and momentum equations [24]. Heat and mass transfer equations are not considered.

- Continuity equation
  (1) $$\frac{\partial \left(\alpha_{k}\right)}{\partial t}+\nabla \left(\alpha_{k}{\bar{u}}_{k}\right)=0$$
- Momentum equation
  (2) $$\frac{\partial \left(\alpha_{k}{\bar{u}}_{k}\right)}{\partial t}+\nabla \left(\alpha_{k}{\bar{u}}_{k}{\bar{u}}_{k}\right)+\nabla \left(\alpha_{k}{\bar{R}}_{k}^{\text{eff}}\right)=-\frac{\alpha_{k}}{\rho_{k}}\nabla p+\alpha_{k}g+\frac{{\bar{M}}_{k}}{\rho_{k}}$$
  (3) $$\alpha_{k}=\frac{V_{k}}{V}$$
  (4) $${\bar{R}}_{k}^{\text{eff}}=-v_{k}^{\text{eff}}(\nabla {\bar{u}}_{k}+\nabla {\bar{u}}_{k}^{T}-\frac{2}{3}I\nabla {\bar{u}}_{k})+\frac{2}{3}Ik_{k}$$

Modeling of particle motion in the liquid phase includes interfacial momentum exchange between the two phases. The major forces exerted on the particle are responsible for drag, lift and virtual mass force. The sum of momentum rates resulting from these forces sets the total interfacial momentum *M_k_* [24].

(5) $${\bar{M}}_{k}=M^{D}+M^{L}+M^{V}$$

The drag force is caused by the relative motion between phases and defined with:

(6) $$M^{D}=\frac{3}{4}\frac{\rho_{c}}{d_{k}}\alpha_{k}C_{D}|u_{r}|u_{r}$$

The drag coefficient calculated according to [25]:

$$C_{D}=\frac{24}{Re}(1+0.15Re^{0.687})$$

The lift force arises due to the shear flow and acts perpendicular to the relative velocity *u_r_* with *C_L,α_* = 0.5. It is given by [26] as follows:

(7) $$M^{L}=C_{L,\alpha }\rho_{c}\alpha_{k}u_{r}\times (\nabla \times u_{c})$$

The force generated by virtual mass depends on the relative acceleration between the phases.

(8) $$M^{V}=C_{\upsilon }\rho_{c}\alpha_{k}(\frac{Du_{c}}{Dt}-\frac{Du_{\alpha_{k}}}{Dt})$$

$\frac{D}{Dt}$ is the total derivative.

Some other forces like Van der Waals and inter-particle electrostatic forces may arise but their effects on the particles behavior are not considered in this approach. Since it is assumed that the particles are detached from the wall at the beginning of the simulation, adhesive and friction force are also not taken into account.

**Figure 2:**
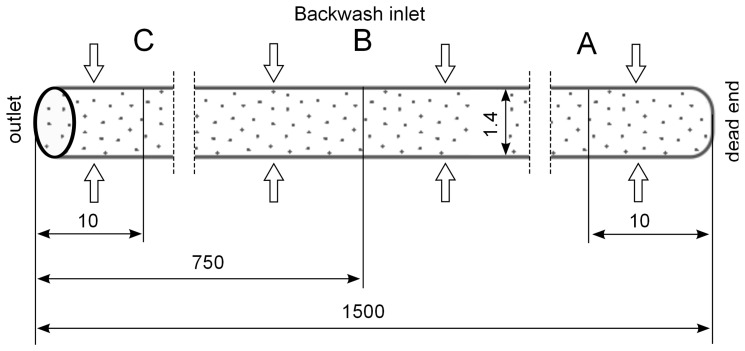
Positions of control lines (A, B, C) for the evaluation of velocity and particle distribution during backwash.

Two types of capillaries operated in dead-end mode are investigated. The first capillary type has an inner diameter and a length of 1.4 mm, 1500 mm, respectively. It is assumed that the particles are homogeneously suspended within the capillary membrane under initial volume fraction *α* of 10% (Figure 2). This model is descritized in 3 millions structured control volumes generated in ANSYS ICEM CFD (Ansys Ltd) where the elements are uniformly distributed across as well as along the computational domain.

**Figure 3:**
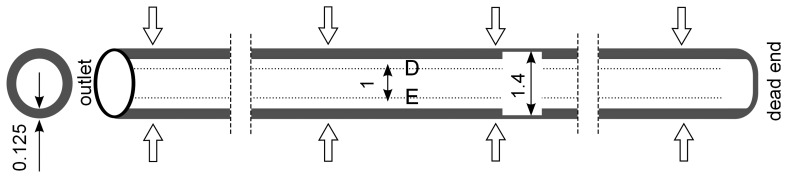
Positions of control lines (*D* = 500 μm, *E* = −500 μm) for the evaluation of velocity and particle distribution during backwash.

**Figure 4:**
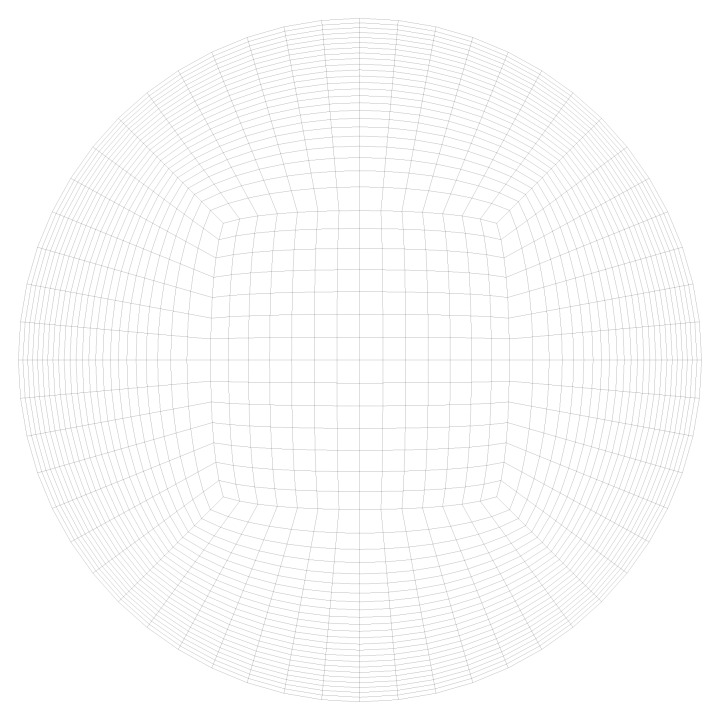
Mesh in the calculation domain.

The second capillary type has an inner diameter of 1.4 mm and a length of 980 mm. In this type the particles are deposited on the inner wall of the capillary forming a ring with thickness of 125 μm and a packing density of 0.6 (Figure 3). Here, 10 millions structured elements are generated with a very fine mesh next to the wall which increases towards the capillary center (Figure 4) and the height of the smallest element is 2.3 μm.

#### 2.2.3. Initial and Boundary Conditions

The initial condition of the particles in the capillary describes (1) a homogeniously distributed suspension at rest and (2) particles deposited in the form of a cake layer. Various boundary conditions have been implemented in numerical simulations in order to examine in details the effects of the operation conditions on performance of backwash process and to improve membrane cleaning efficiency. The diameter of the particles varies between 5 and 20 μm. Moreover, a constant water flux has been chosen between 100 and 400 L m^−2^ h^−1^ and applied at inlet. Dead-end is defined as closed end of the capillary with wall properties and no-slip condition. The ambient pressure is given at outlet. During numerical calculations a convergence criterion of 10^−6^ is set to guarantee a converged solution. The time step control is realized with an adaptive time stepping procedure assumed a maximum Courant number of 0.8. The stable calculations run with the time step in the range of 10^−5^ s. Gaussian linear scheme is implied for the approximation and interpolation. First order, bounded and implicit time discretisation scheme is specified for the solution.

## 3. Numerical Results

Transient simulations of the particle accumulation in the membrane capillary are carried out at different operation parameters. The computational approach developed in this study and the evaluation of the results are predicted in term of particle volume fraction in the cell. Moreover, the diagrams and figures presented in dimensionless form defines r* in the abscissa which denotes the dimensionless diameter of the capillary where zero is on the axis.

The simulation results are evaluated by inserting control lines A, B and C inside the capillary at a distance of 10 mm from the dead-end, in the middle and 10 mm from the outlet across the capillary, respectively (Figure 2) and inserting control lines D and E at a distance of 500 μm from the axis along the capillary (Figure 3).

A parabolic velocity profile is reached in a very short time of the simulation inside the capillary where the flow changes rapidly from radial at dead-end to axial at the outlet. The axial component of the velocity becomes dominant along the capillary compared with the radial velocity component. Due to a constant flux condition at the inlet, the flow rate increases along the membrane and the maximum value is recorded at the outlet.

**Figure 5:**
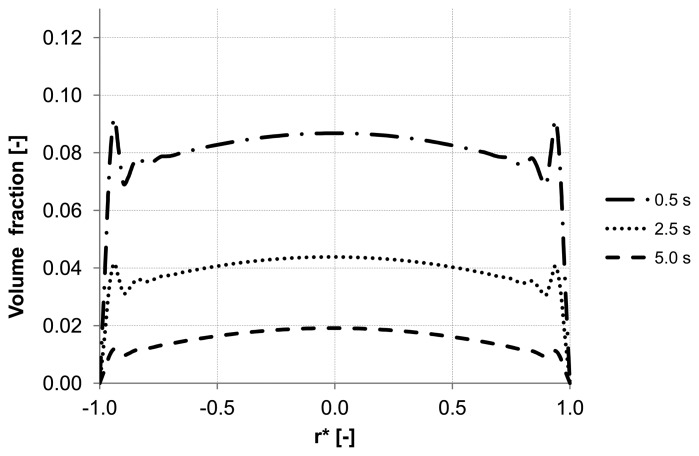
Particle distributions along control line A with backwashing flux of 300 L m^−2^ h^−1^ during 5 s (homogeneous initial distribution, particle diameter of 20 μm).

**Figure 6:**
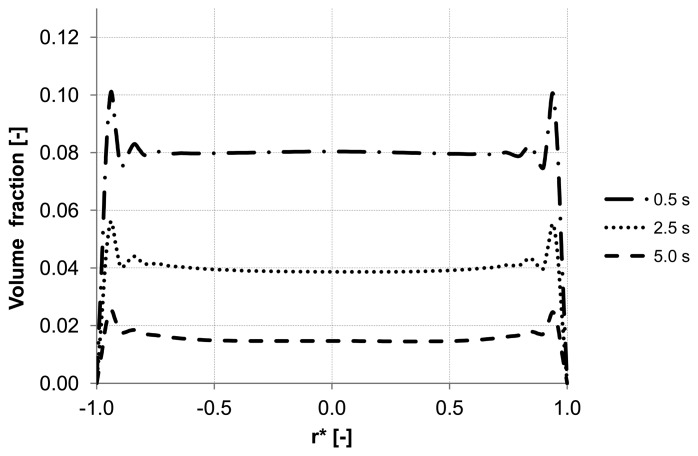
Particle distributions along control line B with backwashing flux of 300 L m^−2^ h^−1^ during 5 s (homogeneous initial distribution, particle diameter of 20 μm).

**Figure 7:**
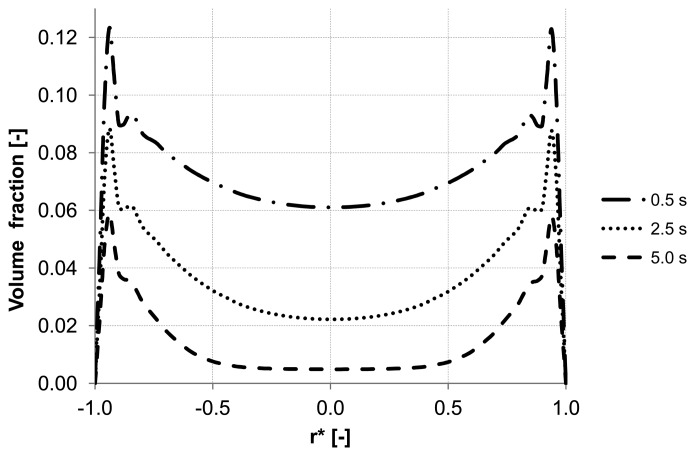
Particle distributions along control line C with backwashing flux of 300 L m^−2^ h^−1^ during 5 s (homogeneous initial distribution, particle diameter of 20 μm).

Analyzing the particle distribution along the three control lines (A, B, C) over the time as illustrated in Figure 5, Figure 6 and Figure 7, a heterogeneous particle distribution inside the membrane could be observed. The particles move towards the wall where their accumulation occurs. Thus, the volume fraction increases next to the wall compared with the values around the axis and decreases over the time when the particles are flushed out of the capillary (Figure 7). The main force responsible for the particle distribution in the membrane is the lift force. It determines the flow patterns and particle enrichment areas in the capillary. At the dead-end control line A, where a relatively small axial velocity gradient is observed the lift force is negligible as Figure 5 shows. However, this force gets more significant with increasing the volume flow rate since the axial velocity gradient of the continuous phase becomes larger (Figure 6). The middle control line B illustrates a lateral migration of particles depending on the Reynolds number *Re* and velocity gradient.

**Figure 8:**
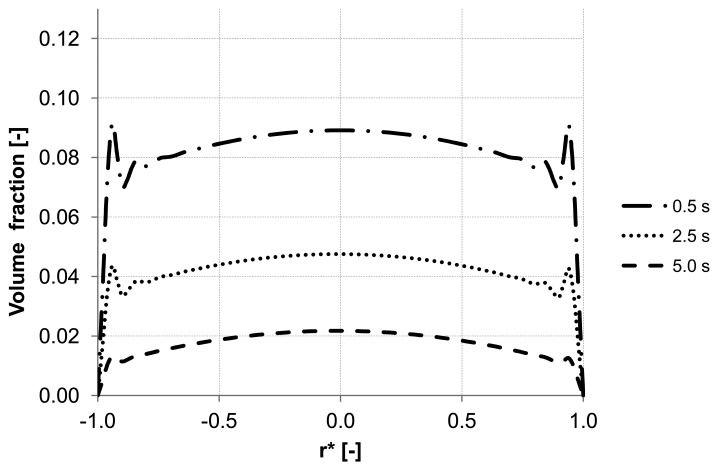
Particle distributions along control line C with not considered lift force and backwashing flux 300 L m^−2^ h^−1^ during 5 s (*cf*. Figure 7).

To emphasize the important role of the lift force, numerical simulation of the backwash process was carried out while the lift force was not considered (Figure 8). Comparing Figure 7 and Figure 8, where the particle distribution at control line C is simulated with and without lift force, respectively, a considerable difference is observed. In contrast to Figure 8, the particles are driven towards the wall due to the effect of the lift force in Figure 7. The distribution of the particles at control line C without lift force is similar to that distribution shown in Figure 5 at control line A where the lift force is very small due to a small axial velocity gradient. This comparison leads to the conclusion that neglecting the lift force causes a similar distribution of the particles at all control lines (A, B, C) in the capillary.

Virtual mass force can be dropped out of the calculation since the relative acceleration between the phases is negligible due to rather small particle diameter (smaller than 20 μm) and almost similar density of both phases.

**Figure 9:**
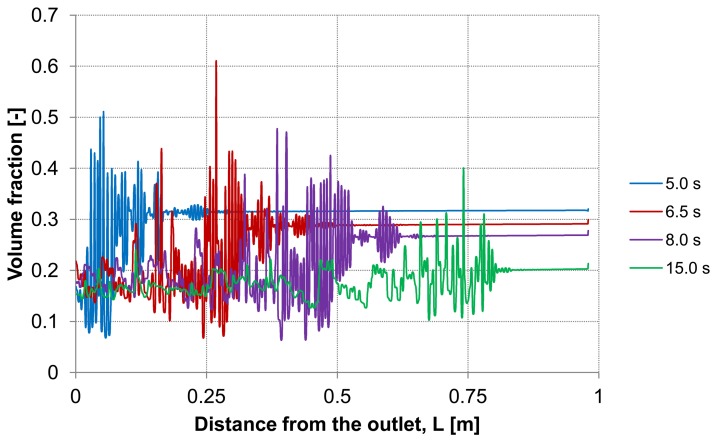
Particle distribution at control line D with backwashing flux of 300 L m^−2^ h^−1^, (deposited layer as initial distribution, particle diameter of 7 μm).

**Figure 10:**
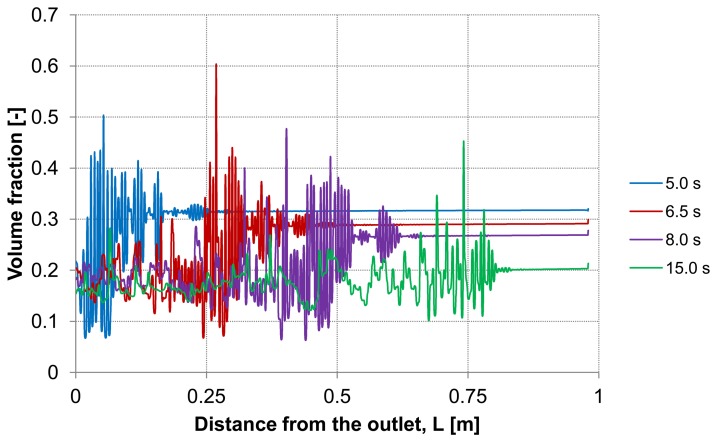
Particle distribution at control line E with backwashing flux of 300 L m^−2^ h^−1^, (deposited layer as initial distribution, particle diameter of 7 μm).

Considering the second capillary with a deposited layer, Figure 9 and Figure 10 demonstrate the particle distribution at the control lines D and E for different backwash times ranging between 5 and 15 s. The gravity effect is pretty small due to insignificant density difference between the water and considered particles. Nevertheless, the particle distribution in lower and upper areas of the capillary shows a slight deviation from the symmetry on the control lines. As expected, a higher value of volume fraction can be seen in the lower part of the capillary. The particles behavior causes heterogeneity inside the capillary.

This heterogeneity begins next to the capillary outlet and propagates towards dead-end causing a critical increase of the particles volume fraction at certain positions. The tendency to form plugs inside the capillary has a considerable effect on backwash process. For instance, permeability, membrane resistance and energy consumption are influenced by the presence of additional obstacles.

The dilution of the detached layer depending on the distance from the outlet side for different backwash times (lines D and E in Figure 3) are shown in Figure 9 and Figure 10, respectively.

At both, the upper control line D and the lower line E, consistent particles distribution is observed including some temporary peaks which refer to an enrichment of particles. The volume fraction of the particles at these peaks reaches a similar value of the initial packing density of the deposited layer. These peaks are first formed at the outlet and shifted during continuous operation of the backwash process in dead-end direction. The particle enrichment areas may lead to a probable formation of plugs inside the capillary which are accordingly moving towards dead-end.

### 3.1. Effect of the Particle Size

Further analysis of the backwash process was performed by considering different sizes of the suspended particles. The water flux of 300 L m^−2^ h^−1^ was constant along the inlet. Three different particle sizes were simulated 5 μm, 10 μm and 20 μm which had the same material properties.

**Figure 11:**
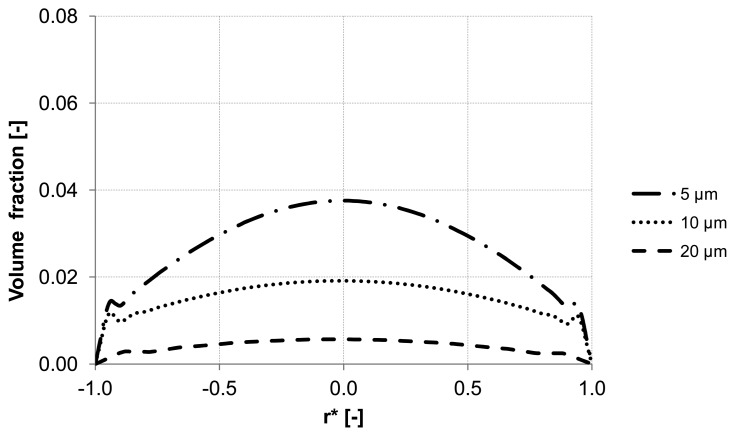
Particle distribution along control line A with different particle size after 5 s (homogeneous initial distribution, backwashing flux of 300 L m^−2^ h^−1^.

**Figure 12:**
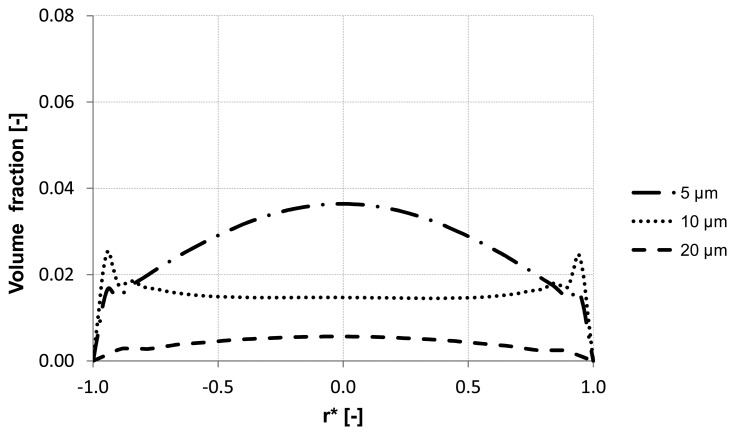
Particle distribution along control line B with different particle size after 5 s (homogeneous initial distribution, backwashing flux of 300 L m^−2^ h^−1^.

**Figure 13:**
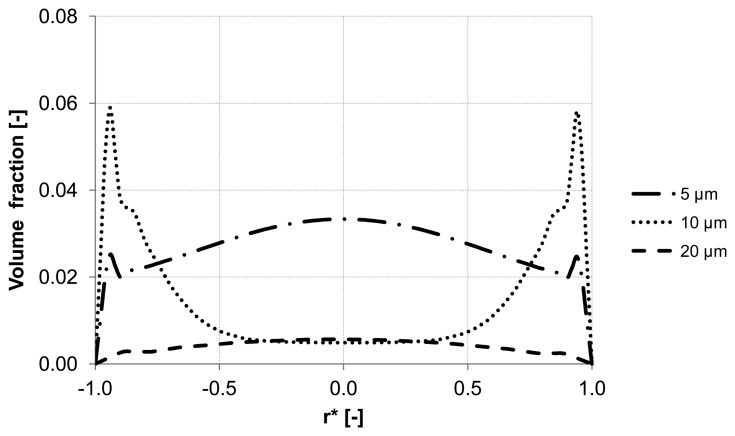
Particle distribution along control line C with different particle size after 5 s (homogeneous initial distribution, backwashing flux of 300 L m^−2^ h^−1^.

The evaluation of the particle distribution along the three control lines (A, B, C) mentioned previously at different backwash times shows different particle behavior in the membrane capillary according to the particle size. For instance, particles with a diameter of 20 μm response quickly to the continuous phase and flushed out without causing accumulation areas inside the membrane. Whereas particles with the size of 5 μm and 10 μm reveal heterogeneous particle enrichment areas in the axial region and close to the wall where accumulation areas are developed. Additionally, a significant enrichment of particles is observed close to the wall of the membrane. The distribution of the volume fraction for different particle sizes along control lines (A, B, C) is illustrated in Figure 11, Figure 12 and Figure 13.

### 3.2. Effect of the Backwash Flux

**Figure 14:**
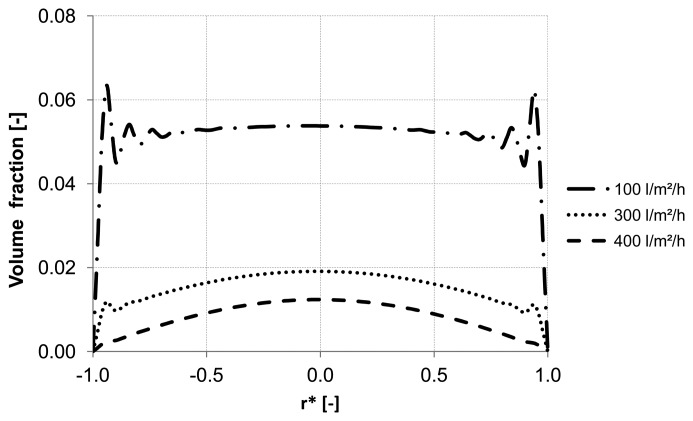
Particle distribution along control line A with various backwashing fluxes after 8 s (homogeneous initial distribution, particle diameter of 10 μm).

**Figure 15:**
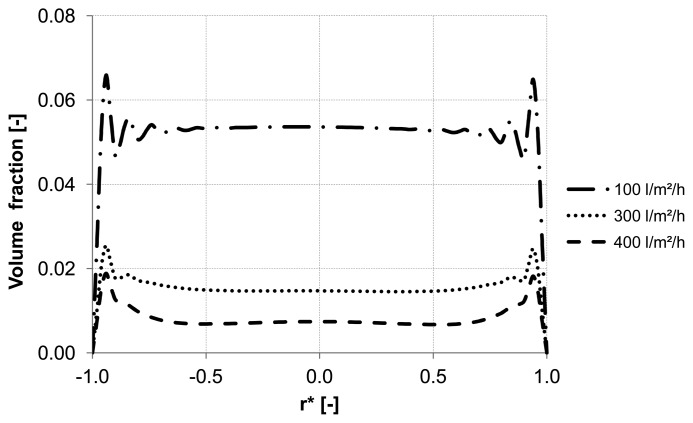
Particle distribution along control line B with various backwashing fluxes after 8 s (homogeneous initial distribution, particle diameter of 10 μm).

**Figure 16:**
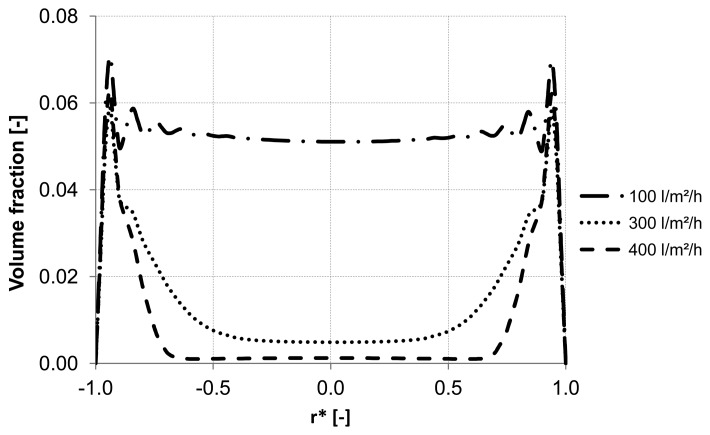
Particle distribution along control line C with various backwashing fluxes after 8 s (homogeneous initial distribution, particle diameter of 10 μm).

Different backwash fluxes have been investigated in order to cover a wide range of operation parameters implemented in industrial water treatment plants. An effective cleaning process is accompanied by an optimal and easy adjustable flux, which flushes off the particles and prevents the formation of plugs. Based on homogeneous particle distribution shown in Figure 2, the effect of backwash flux was investigated while other operation conditions were kept constant. The simulation results highlight the fact that backwash flux is a significant parameter in determining the particle accumulation areas inside the membrane. The analysis of the diagrams Figure 14, Figure 15 and Figure 16 at the control positions A, B and C shows a decrease of the volume fraction of particles with increasing mass flow rate. Moreover, for a flux of 100 L m^−2^ h^−1^ the particles are driven to the outlet without considerable lateral migration. The motion across the streamlines and the corresponding behavior of the suspended particles are caused by the relative velocity which at low Reynolds number points parallel to capillary axis. Thus, the lift force tends to drift away accumulated particles to the capillary outlet.

Figure 14, Figure 15 and Figure 16 illustrate the volume fraction of particles across the capillary at the three control lines (A, B, C in Figure 2) over backwash time. The same figures also demonstrate a faster washing out of particles from the capillary axis when the capillary is operated with a backwash flux of 300 L m^−2^ h^−1^ and 400 L m^−2^ h^−1^.

**Figure 17:**
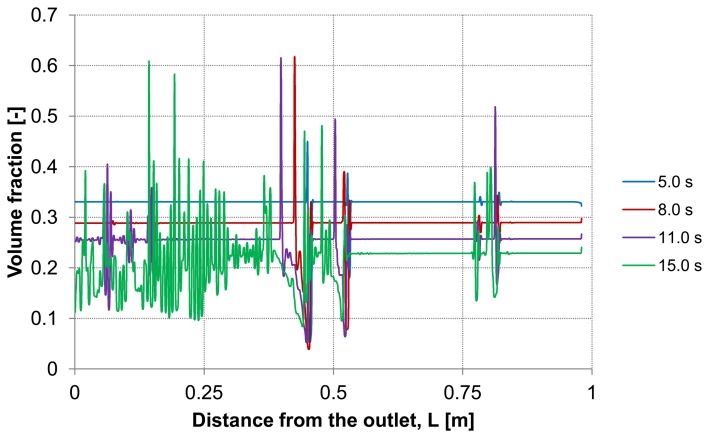
Particle distribution at control line D with backwashing flux of 100 L m^−2^ h^−1^, (deposited layer as initial distribution).

**Figure 18:**
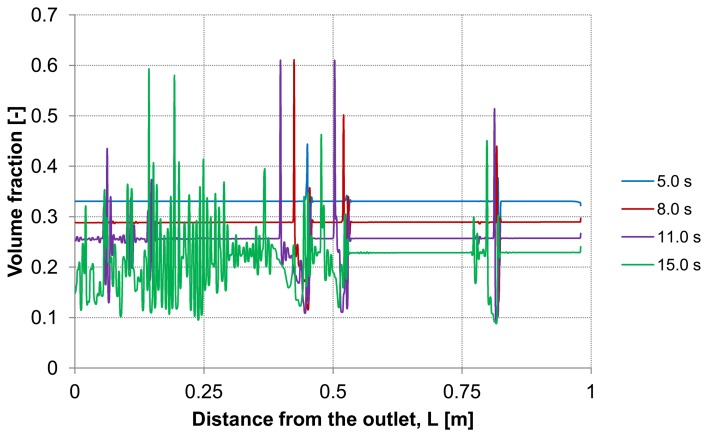
Particle distribution at control line E with backwashing flux of 100 L m^−2^ h^−1^, (deposited layer as initial distribution).

In order to investigate the real conditions for the backwash process, initial particle distribution delivered by the experimental data (Figure 3) was adjusted and correlated to different fluxes. The evaluation of the simulation results confirms a relatively smooth motion of the particles with less peaks and turbulent behavior inside the capillary at low flux (Figure 17 and Figure 18).

## 4. Discussion and Conclusion

Numerical simulations were performed in order to find operation parameters for membrane backwash suitable for quantitative process description, which reflects on a better membrane cleaning performance. This study considered the effect of the lift force on the behavior of the particles as it shifts the suspended particles into wall direction due to the large velocity gradient close to the wall. It was found that this force has the dominant influence in enhancing the lateral migration of the particles in wall direction and in determining the enrichment areas and possibly the formation of plugs inside the membrane. It was proven that the lateral migration of particles and the location of its maximum volume fraction are strongly influenced by the particle size and the inlet water flux. For large particle size, the backwash process maybe favorable because these particles are distributed more homogeneously over the cross section inside the capillary and are washed out of the capillary relatively early. In contrast to the small particles which tend to cause enrichment areas where the axial velocity component is getting larger compared to the radial velocity component. Increasing backwash inlet flux provides fast destruction of the deposited layer and more heterogeneity of particle distribution inside the capillary whereas, decreasing the inlet flux causes a smooth motion of particles, which may avoid the high possibility of the membrane clogging. For the studied process, the virtual mass force becomes insignificant when the particle size is in microscale.

## Nomenclature

$\frac{D}{Dt}$: substantive derivative
${\bar{R}}_{k}^{\text{eff}}$: Reynolds stress tensor (m^2^ s^−2^)
*C_D_*: coefficient of interphase drag force
*C_υ_*: coefficient of interphase virtual mass force
*C_L,α_*: coefficient of interphase lift force
*d*: particle diameter (m)
*g*: gravitational acceleration (m s^−2^)
*I*: identity tensor
*k*: turbulent kinetic energy (J kg^−1^)
*M^D^*: drag momentum rate (kg m^−2^ s^−2^)
*M^L^*: lift momentum rate (kg m^−2^ s^−2^)
*M^V^*: virtual mass momentum rate (kg m^−2^ s^−2^)
*M_k_*: averaged interphase momentum rate acting on phase *k* (kg m^−2^ s^−2^)
*Re*: Reynolds number
*u*: velocity (m s^−1^)
*u_r_*: relative velocity (m s^−1^)
*V*: volume (m^3^)
*α*: volume fraction
*ν_eff_*: effective kinematic viscosity (m^2^ s^−1^)
*ρ*: density (kg m^−3^)
*c*: subscript refers to continuous phase
*k*: subscript refers to the phase
